# The Maternal Postpartum Quality of Life Instrument (MPQOL-I): development and psychometric evaluation in an exploratory sequential mixed-method study

**DOI:** 10.1186/s12884-022-04900-y

**Published:** 2022-07-19

**Authors:** Tahereh Mokhtaryan-Gilani, Nourossadat Kariman, Hamid Sharif Nia, Mahbobeh Ahmadi Doulabi, Malihe Nasiri

**Affiliations:** 1 Midwifery and Reproductive Health Department, School of Nursing and Midwifery, Shahid Beheshti University of Medical Sciences, Tehran, Iran; 2 Midwifery and Reproductive Health Research Center, Midwifery and Reproductive Health Department School of Nursing and Midwifery, Shahid Beheshti University of Medical Sciences, P.O. Box: 1996835119, Vali-Asr Avenue, Vali-Asr and Neiaiesh Highway Intersection, Opposite Rajaee Heart Hospital, Tehran, Iran; 3 School of Nursing and Midwifery, Mazandaran University of Medical Sciences, Sari, Iran; 4 School of Nursing and Midwifery, Shahid Beheshti University of Medical Sciences, Tehran, Iran

**Keywords:** Quality of life, Instrument development, Psychometric assessment, Postpartum period, Validity, Reliability

## Abstract

**Background:**

“ Postpartum quality of life” refers to women’s satisfaction of their position in life, based on cultural status, expectations, values, attitudes, goals, and living standards. Hence the need to pay attention to more specific dimensions of quality of life in the postpartum period is being sensed. This study was conducted to develop the Maternal Postpartum Quality of Life Instrument (MPQOL-I) and assess its psychometric properties.

**Methods:**

This methodological study was conducted in 2019–2020. This exploratory, sequential mixed-method study was conducted in two phases. The first phase is MPQOL-I development and the second phase is psychometric evaluation of the developed scale. In the quantitative (psychometric evaluation) phase, face, content, construct, convergent, and discriminant validity and reliability of the scale were tested.

**Results:**

In this study, 5 factors were extracted from items through exploratory factor analysis: (1) received support, (2) sexual relations, (3) bonding with newborn, (4) breastfeeding and newborn care, and (5) the transition period. These factors accounted for 53.26% of the total variance. The results of the confirmatory factor analysis suggested the goodness-of-fit indices was acceptable. Furthermore, the internal consistency and composite reliability indices of factors were greater than 0.7.

**Conclusion:**

The sixteen-item Persian language MPQOL-I is a valid and reliable instrument for postpartum quality of life assessment. It includes items from different aspects of postpartum quality of life and can be used for the early diagnosis of impaired postpartum quality of life. Further studies are needed to assess the psychometric properties of MPQOL-I in different cultures and communities.

## Background

History and Etymology for postpartum from the Latin phrase post partum "after childbirth," from post "after" + partum, accusative of partus "act of giving birth, childbirth," from parere "to give birth to, bring into being" + -tus [1]. Its duration is understandably inexact, but is considered to be between 4 and 6 weeks [2].

During the postpartum period, the body of women returns to its pre-pregnancy physiological and anatomical conditions [3]. This process is associated with many different psychosocial changes and new roles, which may cause challenges for women when adjusting and prioritising within this new context [4]. Moreover, physiological changes, reorganisation of life and interrupted sleep may affect the woman’s quality of life. Inappropriate postpartum adjusting can cause postpartum complications and reduce the maternal quality of life (QOL) [5].

QOL refers to an individual’s perception of life based on the existing cultural conditions, values, attitudes, goals, and standards [6]. According to the World Health Organisation, QOL has six main components: physical health, psycho-emotional status, level of independence, social relationships, spiritual beliefs, and environmental status [7]. QOL also determines life’s positive and negative characteristics and includes satisfaction with physical health, family, education, employment, possessions, financial status, environment, and religious beliefs [7]. QOL is directly affected by the sociocultural context [8]. Factors reducing postpartum QOL include socio-demographic factors [9], inadequate social support, heavy workload, husband’s limited engagement in household affairs [10], financial problems, fatigue [11], postpartum depression [12], sexual dysfunction [13], number of pregnancies, the method of childbirth [10]. and pregnancy-related complications [14]. Reduced postpartum QOL can negatively affect women’s childrearing behaviours and children’s health [9].

Improvement of QOL and health is a main health-related challenge of the twenty-first century [15].

The essential step to develop effective plans for postpartum QOL improvement is a careful assessment of postpartum QOL, [16] such that its assessment turns into an inseparable part of postpartum care [17]. Such assessment can provide reliable data and help in developing effective plans for improving women’s postpartum states [18].

There are limited instruments for postpartum QOL assessment. One of these instruments is the Mother-Generated Index(MGI) which is a subjective self-administered instrument [19]. The difficulty and complexity of implementing this instrument has limited its use [19]. The Maternal Postpartum QOL questionnaire [20] and the Postpartum QOL questionnaire [5] are two other instruments for postpartum QOL assessment. To the best of our knowledge, none of these instruments include dimensions of women’s satisfaction with postpartum transition period and mother-infant bonding. Therefore, comprehensive but straightforward instruments are needed for careful the postpartum QOL assessment. The present study was conducted to fill this gap. The study aimed to develop the Maternal Postpartum Quality of Life Instrument (MPQOL-I) and assess its psychometric properties in Tehran (Iran).

## Methods

This methodological study was conducted from June 2019 to April 2020 using an exploratory sequential mixed-method design on women living in Tehran, Iran. The study consisted of two main phases: MPQOL-I development and MPQOL-I psychometric evaluation.

### Phase 1: MPQOL-I development

The primary draught of MPQOL-I was developed using the steps recommended by Waltz et al. [21]. Initially, a qualitative study was conducted using the conventional content analysis approach recommended by Graneheim and Lundman [22] in order to explore the concept of postpartum QOL and its dimensions. Participants were postpartum women who were recruited purposefully and with maximum variation in terms of their age, educational level, financial status, type of childbirth, type of infant feeding, number of children, and infant’s gender and age. Inclusion criteria were mothers over eighteen years of age with a healthy infant aged 1–6 weeks and no severe physical or mental disorder such as depression. Participants completed the Edinburgh Postnatal Depression questionnaire, which globally used for postpartum depressive symptoms screening [23], before the interview and their scores were extracted. Finally, individuals with a score of 12 or less were included in the study. Data were collected through semi-structured interviews continued up to data saturation and were analysed through conventional content analysis. The items of MPQOL-I were developed using the findings of the qualitative study and the existing literature on postpartum QOL. Items were revised in a panel of experts.

### Phase 2: Psychometric assessment

**Fig. 1:**
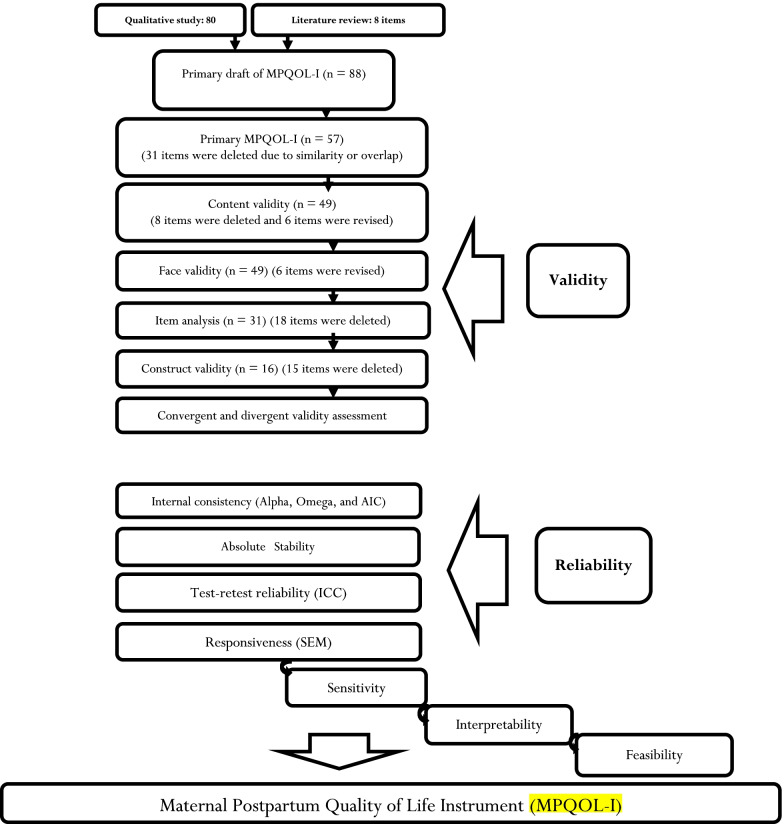
The flow chart of MPQOL-I development and psychometric assessment

In this phase, the psychometric properties of MPQOL-I, consisting of face, content and construct validity, and reliability, were assessed. Figure 1 illustrates the details for each step of MPQOL-I psychometric evaluation.

#### Assessment of content validity

For the qualitative assessment, they were asked to comment on the items’ difficulty, wording, grammar, and comprehensibility. MPQOL-I was revised according to their comments.

For the quantitative assessment, the experts were asked to comment about the essentiality and the relevance of each item based on three- and two-point scales, respectively [24]. Essentiality rating scores were used to calculate content validity ratio (CVR) with this formula: $CVR=\left({N}_{e}-N/2\right)/\left(N/2\right)$. Based on Lawshe’s table and the number of experts, items with CVR values of 0.42 and above were considered appropriate [24]. On the other hand, relevance rating scores were used to calculate each item’s content validity index (I-CVI) by dividing the number of experts who had given that item the score of 3 or 4 by the total number of experts. The items with CVI values greater than 0.79 were considered appropriate, those with CVI values equal to 0.7–0.79 were revised, and the items with CVI values less than 0.7 were excluded [25]. Modified Kappa statistic was also calculated for each item, and items with Kappa values greater than 0.7 were considered appropriate [26].

#### Assessment of face validity

Twenty postpartum women qualitatively and quantitatively assessed the face validity of MPQOL-I. For qualitative face validity assessment, women commented on the comprehensibility of the items and responded to the four questions of the COSMIN methodology regarding the face validity of the items [27]. The four questions participants needed to answer were: “was there any difficulty in comprehending the items?”, “Was there any item you did not want to answer?”, “Was there any topic related to your childbirth experience which had not been addressed in the instrument?”, “Was there any item in the instrument which was not related to the postpartum period?”.

The quantitative assessment of face validity was performed by calculating the items’ impact scores. The same women were asked to rate the importance of each item on a five-point scale. The scores were ranged from 1(“Unimportant”) to 5 (“Very important”). Then, each item’s impact score was calculated by multiplying the frequency of participants who scored that item 4 or 5 by the mean importance score of that item. The items with impact scores higher than 1.5 were considered appropriate [24, 26].

### Item analysis

After the assessment of face and content validity, 31 eligible women completed MPQOL-I. Their data were used to calculate the Cronbach’s alpha of the instrument and its items. Considering coefficients of correlation between item scores and total MPQOL-I score and the changes of total Cronbach’s value with the exclusion of each item, poor items were determined and excluded. Moreover, items with a difficulty index less than 0.2 or more than 0.9 were interpreted as very simple and very difficult, respectively, and excluded [28].

#### Assessment of construct validity

Construct validity was assessed through exploratory factor analysis (EFA), confirmatory factor analysis (CFA), and convergent and discriminant validity assessments. The sample size for factor analysis was calculated based on the 5–10 participants per item rule [29]. In total, 700 postpartum women were invited to the study through personal telephone contact. Subsequently, 390 postpartum women were purposefully recruited from twenty healthcare centres in Tehran, Iran, to complete MPQOL-I for exploratory factor analysis. Inclusion criteria were mothers over eighteen years of age with a healthy infant aged 1–6 weeks, ability to read and write, without formal diagnosis physical disability or mental disorder, and a score less than 13 for the Edinburg Postnatal Depression Scale. Participants completed MPQOL-I and Edinburgh Postnatal Depression questionnaire either online or in person. Exploratory factor analysis was performed using the SPSS software (v. 26.0). Sample adequacy was determined through the Kaiser–Meyer–Olkin and Bartlett’s tests. A Kaiser–Meyer–Olkin value greater than 0.7 was interpreted as an adequate sample [30]. Latent factors were extracted through the maximum likelihood estimation with Promax rotation and Horn’s parallel analysis [31]. Factor loading values greater than 0.3 and eigenvalues greater than 1 were considered appropriate [32, 33].

For confirmatory factor analysis, 201 eligible women were purposefully recruited to complete MPQOL-I either online or in person. The population, setting, sampling method, sample size calculation, and inclusion criteria for confirmatory factor analysis were the same as exploratory factor analysis [34]. Confirmatory factor analysis was performed through the AMOS software (v. 24). Model fitness was assessed using the following fit indices (Table 2) [34].

Convergent and discriminant validity were assessed through Fornell and Larcker criterion and by calculating average variance extracted (AVE), maximum shared squared variance (MSV), and composite reliability (CR). An AVE value greater than 0.5 shows acceptable convergent validity, and an average variance extracted value greater than maximum shared squared variance shows acceptable discriminant validity [34].

#### Assessment of reliability

The reliability of MPQOL-I was assessed by calculating Cronbach’s alpha, McDonald’s omega, Composite reliability (CR), average inter-item correlation (AIC), and coefficient H [35]. Stability was assessed by calculating the test–retest intraclass correlation coefficient (ICC), which was estimated through the two-way mixed effects and with a confidence level of 95%. Moreover, standard error of measurement (SEM) and minimum detectable change (MDC) were calculated as a part of absolute stability and criteria for responsiveness [36]. Standard error of measurement was calculated through the *SEM* = *SD*√1-*ICC* formula, where *SD* was the standard deviation of the sum values obtained in the test and the retest phases. Minimum detectable change was also calculated through the $MDC=SEM\times Z\times \sqrt{2}$ formula, where *Z* was 1.96 and the level of confidence was 0.95. The relative amount of random measurement error was also calculated through this formula, $MDC\%=\left(MDC/Mean\right)\times 100$. An MDC% value of less than 30% is acceptable, and a value of less than 10% is excellent [36, 37].

### Sensitivity

Sensitivity was assessed through hypothesis testing [38]. The hypothesis was “Postpartum QOL has a significant relationship with the type of infant feeding.”. This hypothesis was tested using the one-way analysis of variance.

### Interpretability

Interpretability was assessed by calculating minimal importance change (MIC) through the $MIC=0.5\times SD of the \Delta score$ formula. A minimal importance change greater than minimum detectable change confirms interpretability [39, 40].

### Feasibility

We attempted to use robust methods for psychometric assessment and kept only the most important items to develop an instrument with an acceptable number of items and a short response time [39].

### Scoring

MPQOL-I items were scaled based on a five-point Likert scale as follows: 1: “None”; 2: “Little”; 3: “Moderate”; 4: “Much”; and 5: “Very much.” Items 12, 25, and 28 were reversely scaled. The possible total score of the instrument is 16–80, with higher scores showing better QOL.

### Outliers, normal distribution of the data, and missing data

In the final analysis, the frequency of the missing data was zero because the online version of the instrument featured compulsory items, and the missed data in the questionnaire of those participants who answered the instrument in-person were collected through making phone calls and asking for their answers to the missed items. The normal distribution of the data was assessed using both univariate and multivariate distribution testings. Multivariate outliers were determined through the Mahalanobis d-squared (*P* < 0.001), and multivariate normality was tested using the Mardia coefficient. A Mardia coefficient value of less than 8 was considered acceptable [41].

The Ethics Committee of Shahid Beheshti University of Medical Sciences, Tehran, Iran, approved this study. Necessary permissions for the study were obtained from the authorities of the university. Participants received clear information about the study aim and the confidentiality of their data and provided informed consent for participation. Free online midwifery counselling was offered to participants for one year in order to increase the response rate.

## Results

### The findings of the MPQOL-I development phase

Sampling was continued until data saturation. The study was saturated with 16 interviews, then 4 more interviews were conducted to confirm data saturation, and finally, with 20 interviews, the qualitative analysis was completed.

The conventional content analysis of the interviews with twenty postpartum women resulted in the development of 1009 primary codes and reduced to 113 final codes in 41 subcategories, sixteen main categories, and six main themes. Based on the qualitative study phase, postpartum QOL was defined as a relative and multidimensional concept affected by women’s perceptions and experiences of the support received from husband, family, and cyberspace, maternal and neonatal psycho-emotional conditions, maternal health status, breastfeeding and neonatal care status, socioeconomic status, and change in the rhythm of life according to maternal roles. Primary draught of MPQOL-I was revised in a panel of experts, which consisted of the study authors, and the primary 57-item MPQOL-I was developed.

### The findings of the MPQOL-I psychometric assessment phase

#### Assessment of content validity

At first, 20 qualified specialists (ten reproductive health specialists, two instrumentation and methodology specialists, two obstetricians, two psychologists, one nutritionist, two midwives, and a nurse) were surveyed, and the experts’ suggestions were applied to the questionnaire to validate the content quality.

Eight items were omitted due to low CVR or CVI values, and six items were revised. The CVR, CVI, and Kappa values of the remaining 49 items were equal to or greater than 0.37, 0.79, and 0.77, respectively. The average scale-level CVI (S-CVI/Ave) was 0.92.

#### Assessment of face validity

Twenty women in the postpartum period were surveyed for qualitative and quantitative content validity. Some items were revised during the qualitative assessment of face validity. Then, the quantitative assessment of face validity showed that the impact scores of the items were 3.75–5, and hence, none of the items were excluded.

### Item analysis

Thirty-two postpartum women completed the MPQOL-I, and their data were used for item analysis. The total Cronbach’s alpha of the 49-item MPQOL-I was 0.857. Considering the coefficients of correlation between the total score of MPQOL-I and the score of each item, as well as the changes in the total Cronbach’s alpha with the exclusion of each item, eighteen poor items were eliminated, and 31 items remained. All pairwise inter-item correlation coefficients were less than 0.7.

#### Assessment of construct validity

The mean age of the 590 postpartum women who were studied in Exploratory Factor Analysis and confirmatory Factor Analysis was 29.67 ± 5.09 years. The gender of participants’ newborns was male in 52.2% of the cases and female in 47.8% of the cases. The majority of the participants had average financial status (62.7%), high school graduation degree or higher (71%), and had undergone Cesarean Sect. (58.5%). The type of infant feeding was 54% breastfeeding, 37.6% combined breastfeeding and bottle-feeding, and 8.4% bottle-feeding.

In Exploratory Factor Analysis, the Kaiser–Meyer–Olkin Index statistic was 0.807, and Bartlett’s test value was 2302.889 (P < 0.001). During Exploratory Factor Analysis, fifteen more items were removed, and the remaining sixteen items were loaded on five factors which explained 53.26% of the total variance. Horn’s parallel analysis also extracted the same five factors. These five factors were labelled received support, sexual relationship, bonding with newborn, breastfeeding and newborn care, and the transition period (Table 1).

**Table 1:** The results of Exploratory Factor Analysis of MPQOL-I

Factors	Items	Factor loading	h^2^	% of variance	Eigen values
**Perceived support**	Q2: How much does your husband pay attention to you?	0.971	0.847	14.40	2.322
	Q3. How well does your husband understand your new statuses?	0.777	0.631		
	Q1: How much does your husband divide your responsibility with a newborn?	0.721	0.510		
	Q16: How affectionate is your relationship with your husband in the postpartum period?	0.506	0.564		
**Sexual relationship**	Q28: How much has emotional distress developed between you and your spouse due to restrictions on postpartum sex?	0.939	0.887	13.19	2.111
	Q25: How troubled are your marital relations in the postpartum period?	0.794	0.636		
	Q12: How sad do you feel about the changes in your relationship with your husband?	0.774	0.626		
**Bonding with newborn**	Q14. How much does having a newborn soothe you?	0.661	0.515	7.92	1.267
	Q13: How happy are you to be a mother?	0.648	0.421		
	Q15. How much strength do you feel when your newborn is calm next to you?	0.641	0.406		
**Breastfeeding and newborn care**	Q18. How much confidence do you have in caring for your newborn?	0.769	0.632	7.39	1.182
	Q20. How skilled are you at caring for your newborn?	0.656	0.408		
	Q21. How much do you think your breast milk is enough for your newborn?	0.401	0.193		
**Transition period**	Q17. How satisfied are you with your friends and relatives?	0.652	0.498	6.86	1.090
	Q11. How satisfied are you with your recreations with your newborn in the postpartum period?	0.644	0.484		
	Q6. How much contact do you have with friends or relatives?	0.508	0.264		

h^2^**= Communality of the variables**

**Fig. 2:**
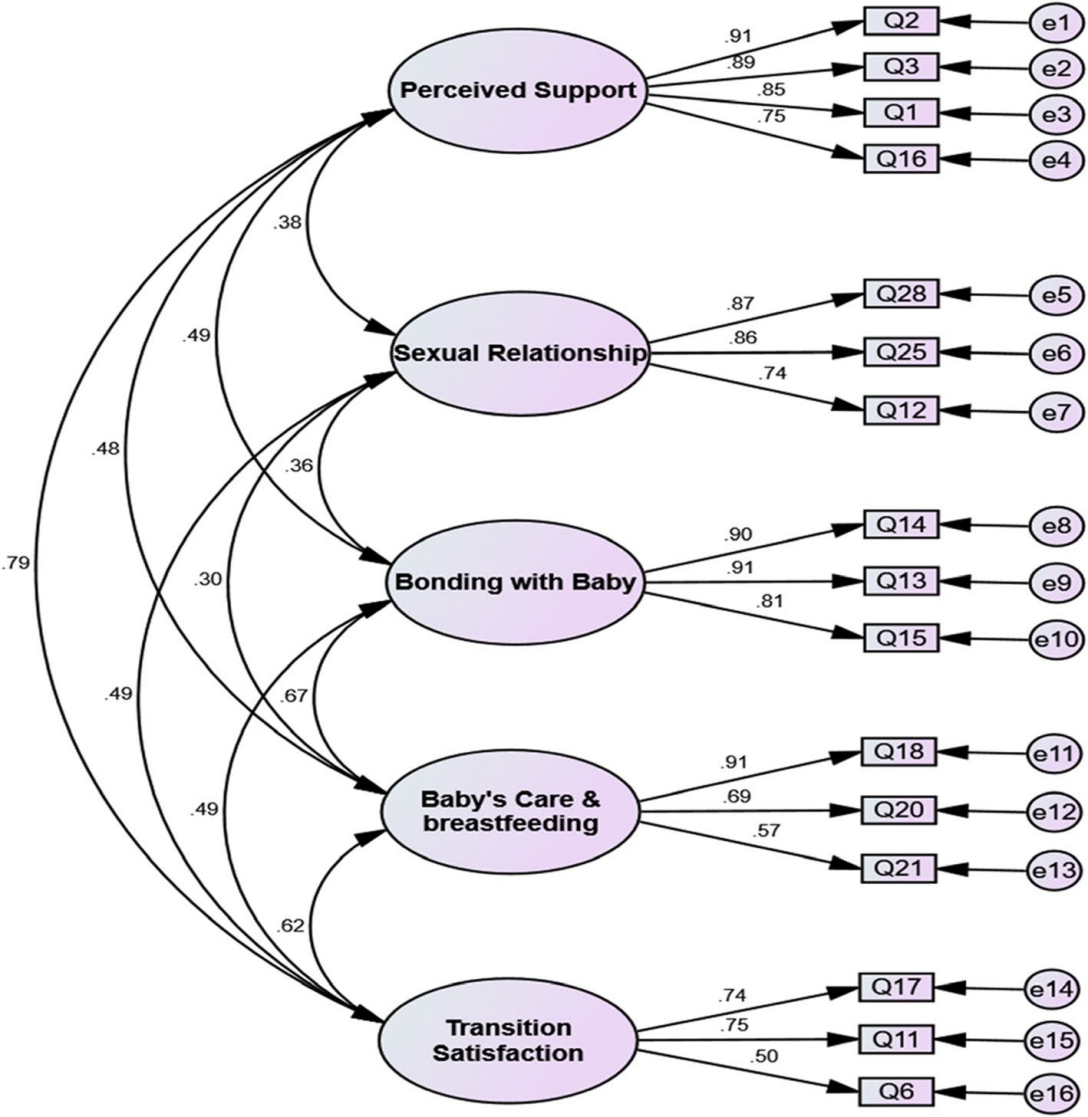
The final structure of MPQOL-I confirmed in CFA

The confirmatory factor analysis showed an acceptable goodness-of-fit. The results of confirmatory factor analysis were shown in Table 2. These findings confirmed the five-factor structure of the sixteen-item MPQOL-I. Figure 2 shows this structure and the coefficients of the pairwise correlations between MPQOL-I items and dimensions.

**Table 2:** Fit model indices of MPQOL-I

Indices Model	χ^2^	df	P value	CMIN/DF	RMSEA	PCFI	PNFI	IFI	CFI
**First-order**	206.087	94	0.001	2.192	0.057	0.738	0.705	0.943	0.942
**Cut off**	–	–	> 0.05	< 3	< 0.1	> 0.5	> 0.5	> 0.9	> 0.9

*χ*^*2*^ Chi-squared, *df* Bartlett test of Sphericity, *CMIN/DF* Minimum Discrepancy Function by Degrees of Freedom Divided, *RMSEA* Root Mean Square Error of Approximation, *PCFI* Parsimonious Comparative Fit Index, *PNFI*Parsimonious Normal fit Index, *IFI* Incremental fit Index, *CFI* Comparative Fit Index

#### Assessment of convergent and discriminant validity

The AVE values of all factors were more than 0.5, and MSV values of all factors were less than AVE values (Table 3). These findings confirmed the acceptable convergent and discriminant validity of MPQOL-I.

**Table 3:** Data on the construct validity and reliability of MPQOL-I

	Construct validity			Reliability				
	AVE	MSV	MaxR (H)	Alpha	Omega	CR	ICC	AIC
Factor 1	0.766	0.034	0.926	0.907	0.911	0.970	0.907	0.712
Factor 2	0.756	0.033	0.878	0.860	0.863	0.964	0.860	0.671
Factor 3	0802	0.223	0.914	0.901	0.905	0.970	0.901	0.756
Factor 4	0.731	0.223	0.862	0.746	0.772	0.949	0.746	0.520
Factor 5	0.860	0.015	0.740	0.700	0.719	0.961	0.700	0.438

*AVE* Average variance extracted, *MSV* Maximum shared squared variance, *Alpha* Cronbach’s alpha, *Omega* McDonald’s Omega, *CR* Composite reliability, *ICC* Intraclass correlation coefficient, *AIC* Average inter-item correlation

#### Assessment of reliability

Internal consistency assessment showed that the Cronbach’s alpha, McDonald’s Omega, CR, and coefficient H values of all factors were higher than 0.7 (Table 3). Moreover, AIC values were 0.438–0.756 (Table 3).

Relative stability assessment showed that the ICC of MPQOL-I was 0.919 (95% CI: 0.865–0.954; *P* < 0.001). Absolute stability assessment also showed that SEM was 1.71. MDC and MDC% values were also 4.72 and 8.69%, respectively.

### Sensitivity

The one-way analysis of variance showed at least one significant difference among women from distinct feeding type groups regarding their postpartum QOL (*P* < 0.001). Post hoc analysis revealed that the QOL of women who bottle feeding their neonates was significantly less than those who breastfed or combined breastfeeding with bottle feeding their neonates (*P* < 0.001).

### Interpretability

MIC and MDC values were 5.8 and 4.73, respectively. The greater value of MIC compared to MDC confirmed the good interpretability of MPQOL-I.

### Feasibility

MPQOL-I has sixteen simple and short items in all essential dimensions of postpartum QOL. The response time of the instrument is 8–10 min in the paper-and-pencil version and five minutes in the online version.

## Discussion

This study was conducted to develop MPQOL-I and assess its psychometric properties. Findings revealed that the sixteen-item MPQOL-I has acceptable validity and reliability and its five main factors are perceived support, sexual relationships, bonding with newborn, breastfeeding and newborn care, and satisfaction with postpartum transition.

In the present study, exploratory factor analysis and extracted latent variables showed valuable results. Under the influence of expectations, goals, values, and standards of the postpartum period, women’s perceptions of their situation in life change fundamentally. For instance, economic issues are not as crucial as “newborn bonding with mother” and “caring and breastfeeding” for the mother in the postpartum period. This study showed that in the first six weeks after delivery, economic issues do not affect the quality of life, and it seems that this is a remarkable and essential finding. On the other hand, the “support received” is the most crucial issue of this period. Also, physical health is less important for the mother than bonding with the baby and the care and breastfeeding in the first six weeks after delivery. Issues related to the baby overshadow the mother so much that she pays less attention to her physical health. The present study showed that, contrary to popular belief that economic issues play a decisive role in the quality of life in this period, appropriate support is influential in increasing the quality of life of mothers and can significantly prompt maternal health.

The received support factor of MPQOL-I has four items of husband’s attentiveness, husband’s understanding of the new conditions, affections of the husband, and husband’s engagement in the newborn care. A former study showed that socio-emotional help and support were among the significant predictors of depression, stress, and QOL among pregnant women [42]. Another study reported a significant relationship between postpartum QOL and husband’s support [43]. Similarly, a study found that spousal support and good marital relationship were significant factors contributing to the improvement of postpartum QOL [43]. Support is a key component of almost all QOL-related instruments such as the Maternal Postpartum QOL questionnaire, the Postpartum QOL questionnaire, and the WHOQOL-BREF [5, 20, 44].

The sexual relations factor of MPQOL-I has three items related to problems in sexual relations, including limitations in sexual intercourse and its relevant physical and mental problems. A study showed that sexual dysfunction in the postpartum period could negatively affect women’s QOL and highlighted that despite their high prevalence and significant effects on marital relationships, sexual problems in the postpartum period are often underreported [45]. Some existing instruments for postpartum QOL assessment include one or more items on sexual relations, while MPQOL-I encompasses an essential dimension on this aspect of QOL.

The bonding with newborn factor of MPQOL-I has three items regarding women’s feelings about motherhood and states of authority and calmness with their newborn. Mother-newborn bonding is highly affected by women’s parental skills and can reduce their stress and improve their QOL. Therefore, quality education should be provided to postpartum women to improve their skills, their bonding with their newborns, and thereby, their QOL [46]. A systematic review revealed a wide knowledge gap regarding the relationship of maternal–fetal attachment and early postpartum bonding with maternal mental health and highlighted the necessity of developing valid instruments for postpartum QOL assessment [47]. MPQOL-I is the only postpartum QOL assessment instrument that includes items on bonding.

The breastfeeding and newborn care factor has three items related to women’s feelings about breast milk adequacy, self-confidence in newborn care, and adequate skills for newborn care. A former study showed that women whose newborns got enough sleep and feeding had better mental health and QOL scores than other women [48]. Breastfeeding is a significant factor contributing to successful mother-newborn bonding [49]. Moreover, women with lower levels of prenatal and postnatal anxiety are able to care for their newborns more effectively, have better QOL, and higher mental health status [49]. Breastfeeding and newborn care are significant factors in postpartum QOL. Hence, they should be addressed in postpartum QOL assessment instruments. However, some of the instruments do not incorporate them as a distinct dimension.

The transition period’s factor has three items regarding maternal satisfaction about the time she spends with her husband, level of recreational activities with the newborn, and the relations with friends and relatives. Childbirth significantly changes a couple’s lives and causes them some stress levels in marital relations [50]. This stress makes women establish stronger relationships with their husbands, families, and friends and achieve higher levels of personal development and maturity [50]. The most important sources of support in the postpartum period are family, friends, and colleagues [51]. Indeed, any factor which improves postpartum satisfaction can improve QOL. Nonetheless, none of the existing postpartum QOL assessment instruments include dimensions of the transition period.

The MDC% value of MPQOL-I was 4.72, which is less than 10% and is interpreted as excellent [36, 37]. The SEM value of MPQOL-I was 1.71.

Unlike other postpartum QOL assessment instruments, MPQOL-I was developed based on almost all criteria of the COSMIN methodology for validity assessment and advanced psychometric assessment methods and had a greater focus on the specific aspects of postpartum QOL. Moreover, the construct validity of MPQOL-I was assessed through EFA, parallel analysis, and CFA, its reliability was assessed through Cronbach’s alpha, test–retest stability, absolute stability, McDonald’s Omega, AIC, CR, and coefficient H, and its sensitivity, responsiveness, interpretability, and feasibility were assessed through different methods. However, the studies about the development and the psychometric assessment of the Mother-Generated Index [52], the Maternal Postpartum QOL questionnaire, and the Postpartum QOL questionnaire [5, 20] just provided information about some aspects of psychometric assessment. Moreover, compared to these instruments, the psychometric properties of MPQOL-I were assessed with more samples of postpartum women. MPQOL-I also has fewer items and a shorter response time compared to the existing postpartum QOL assessment instruments.

It should be noted that a questionnaire on postpartum quality of life has not been developed in Iran, and the need for a comprehensive questionnaire that is compatible with the culture of the Iranian people was felt. Since there were limited specific questionnaires available in the world, it is predicted that this questionnaire will be considered an innovation both in Iran and worldwide. The three tools closest to the present study’s topic were examined in Table 4.

**Table 4:** The measurement characteristics of current postpartum quality of life instruments based on CASMIN classification

Postpartum QoL tools	Tool creator & the year	Feasibility	Interpretability	Sensitivity	Measurement error	Stability	Internal consistency	Structural validity	Criterion validity	Content validity
**MGI**	Symon et al. 2003 [19]	**N**	**N**	**N**	**N**	**N**	N*	**Y**	**Y**	Y*
**MAPP-QOL**	Hill et al. 2006 [20]	**N**	**N**	**N**	**N**	**Y**	**Y**	**Y**	**Y**	**Y**
**PQOL**	Zhou et al. 2009 [5]	**N**	**N**	**N**	**N**	**Y**	**Y**	**Y**	**N**	**Y**
**MPQOL-I**	Mokhtarian et al. 2021 [41]	**Y**	**Y**	**Y**	**Y**	**Y**	**Y**	**Y**	**N﻿**	**Y**

For more advanced evaluations, MPQOL-I has used McDonald’s Omega, AIC, CR, and Maximum Reliability H in addition to the above evaluations. Also, in addition to exploratory factor analysis, confirmatory factor analysis and convergent and divergent validity were used to evaluate the structural validity

Y = yes; N = no

### Strength and limitation

The strength points of the present study were the use of more accurate and reliable tests to assess factor structure and reliability measurement. Moreover, the number of modern Parallel Analysis criteria was determined using SPSS R-Meno Version 2. Moreover, confirmed factor analysis was used to validate the instrument besides to exploratory factor analysis in this study. Despite the strengths of this study, there is limitation as well. This study was conducted in Tehran, its result cannot be generalised to all postpartum Iranian women. Iran is a multi-cultural country, it is recommended to measure this questionnaire in different Iranian cultures.

## Conclusion

The sixteen-item MPQOL-I has acceptable validity and reliability for postpartum QOL assessment. Therefore, it can be used as a short and straightforward instrument for assessing postpartum QOL in different settings (e.g., post-partum clinics in different cultural communities). Data obtained through the application of MPQOL-I can be used to develop appropriate interventions for improving QOL and preventing complications among postpartum women.

### Recommendations

Future studies are recommended to use MPQOL-I for postpartum QOL assessment and measure its psychometric properties in different cultures and communities. Authors encourage the researchers to perform more studies in this regard for longer period of postpartum, such as " from sixth week to sixth month" or "from sixth month to first year".

## Data Availability

Due to privacy guidelines of our university, the datasets used and/or analyzed during the current study are available via contacting the corresponding author on reasonable request.

## Abbreviations

AIC: Average Inter Correlation
AGFI: Adjusted Goodness of Fit Index
AVE: average variance extracted
CFA: confirmatory Factor Analysis
CVR: Content Validity Ratio
CVI: Content Validity Index
CFI: Comparative Fit Index
CR: Composite reliability
CMIN/DF: Minimum Discrepancy Function by Degrees of Freedom Divided
EFA: Exploratory Factor Analysis
EPDS: Edinburgh Postnatal Depression Scale
H: Coefficient H
ICC: intra-class correlation coefficient
IFI: incremental fit Index
I-CVI: Item Content Validity Index
KMO: Kaiser–Meyer–Olkin Index
MPQOL-I: The Maternal Postpartum Quality of Life Instrument
MAPP-QOL: Maternal Postpartum Quality Life Questionnaire
MSV: Maximum Shared Squared Variance
MGI: Mother-Generated Index
NFI: Normed Fit Index
PCFI: Parsimonious Comparative Fit Index
PNFI: Parsimonious Normal fit Index
PQOL: Postpartum Quality of Life
QOL: Quality of Life
RMSEA: Root Mean Square Error of Approximation
SEM: Standard Error of Measurement
SRMR: Standardized Root Mean Square Residual
SCVI/Ave: Scale Content Validity Index/Average

## References

[1] Merriam.Webster Dictionary. Definition of postpartum. 2022. In:https://www.merriam-webster.com/dictionary/postpartum. Accessed 18 Apr 2022.

[2] Cunningham FG, Leveno KJ, Bloom SL, Spong CY, Dashe JS, Hoffman BL (2018). Obstetricia de Williams.

[3] Deniz C, Ayaz S (2014). Factors causing stress in women with babies 0–3 months old and their coping styles. J Psychiatr Ment Health Nurs..

[4] Grylka-Baeschlin S, Meyer T, Lengler L, van Teijlingen E, Pehlke-Milde J, Gross MM (2019). Postnatal quality of life — A content analysis of qualitative results to the Mother-Generated Index. Women and Birth..

[5] Zhou SZ, Wang XL, Wang Y (2009). Design of a questionnaire for evaluating the quality of life of postpartum women (PQOL) in China. Qual Life Res..

[6] Chinweuba AU, Okoronkwo IL, Anarado AN, Agbapuonwu NE, Ogbonnaya NP, Ihudiebube-Splendor CN (2018). Differentials in health-related quality of life of employed and unemployed women with normal vaginal delivery. BMC Womens Health..

[7] McGregor JA, Camfield L, Woodcock A (2009). Needs, Wants and Goals: Wellbeing, Quality of Life and Public Policy. Appl Res Qual Life..

[8] Sadat Z, Abedzadeh-Kalahroudi M, Kafaei Atrian M, Karimian Z, Sooki Z (2014). The Impact of Postpartum Depression on Quality of Life in Women After Child’s Birth. Iran Red Crescent Med J..

[9] Fontenele de Oliveira Mi, Parker L, Ahn H, Lívia Oliveira Catunda H, Braga Rodrigues Bernardo E, Fontenele de Oliveira Ma (2015). Maternal Predictors for Quality of Life during the Postpartum in Brazilian Mothers.. Health (Irvine Calif).

[10] Tungchama F, Piwuna C, Armiya’u A, Maigari Y, Davou F, Goar S (2017). Independent socio-demographic and clinical correlates associated with the perception of quality of life of women with postpartum depressionin North central Nigeria. Int J Psychiatry Clin Pract..

[11] Petrou S, Boulvain M, Simon J, Maricot P, Borst F, Perneger T (2004). Home-based care after a shortened hospital stay versus hospital-based care postpartum: an economic evaluation. BJOG An Int J Obstet Gynaecol..

[12] Papamarkou M, Sarafis P, Kaite CP, Malliarou M, Tsounis A, Niakas D (2017). Investigation of the association between quality of life and depressive symptoms during postpartum period: a correlational study. BMC Womens Health..

[13] Ghazanfarpour M, Khadivzadeh T, Babakhanian M (2016). Investigating the Relationship Between Sexual Function and Quality of Life in Menopausal Women. J Fam Reprod Heal..

[14] Rezaei N, Tavalaee Z, Sayehmiri K, Sharifi N, Daliri S (2018). The relationship between quality of life and methods of delivery: A systematic review and meta-analysis. Electron physician..

[15] Taheri-Kalani F, Mami S, Direkvand-Moghadam A, Kaikhavani S, Delpisheh A (2014). Comparison of the effect of delivery type on the quality of life in women attending to health centers of Ilam and Aivan during 2013. J Shahrekord Univ Med Sci..

[16] Akbarzadeh M, Toosi M, Zare N, Sharif F (2012). Effect of Relaxation Training to Pregnant Mothers on Quality of life and Postpartum Blues. Knowl Heal J..

[17] CADTH (2017). Guidelines for the Economic Evaluation of Health Technologies: Canada.

[18] Bahrami N, Simbar M, Bahrami S (2013). The effect of prenatal education on mother’s quality of life during first year postpartum among Iranian women: A randomized controlled trial. Int J Fertil Steril..

[19] Symon A, MacKay A, Ruta D (2003). Postnatal quality of life: a pilot study using the Mother-Generated Index. J Adv Nurs..

[20] Hill PD, Aldag JC, Hekel B, Riner G, Bloomfield P (2006). Maternal Postpartum Quality of Life Questionnaire. J Nurs Meas..

[21] Waltz CF, Strickland OL, Lenz ER. Measurement in nursing and health research. 4th ed. New York: Springer publishing company; 2010.

[22] Graneheim UH, Lundman B (2004). Qualitative content analysis in nursing research: Concepts, procedures and measures to achieve trustworthiness. Nurse Educ Today..

[23] Cox JL, Chapman G, Murray D, Jones P (1996). Validation of the Edinburgh postnatal depression scale (EPDS) in non-postnatal women. J Affect Disord..

[24] Lawshe CH (1975). A quantitative approach to content validity. Pers Psychol..

[25] Hanh VTX, Guillemin F, Cong DD, Parkerson GR, Thu PB, Quynh PT (2005). Health related quality of life of adolescents in Vietnam: cross-cultural adaptation and validation of the Adolescent Duke Health Profile. J Adolesc..

[26] Polit DF, Beck CT, Owen SV (2007). Is the CVI an acceptable indicator of content validity?. Appraisal and recommendations. Res Nurs Health..

[27] Terwee CB, Prinsen CAC, Chiarotto A, Westerman MJ, Patrick DL, Alonso J (2018). COSMIN methodology for evaluating the content validity of patient-reported outcome measures: a Delphi study. Qual Life Res..

[28] Thorndike RM, Cunningham GK, Thorndike RL, Hagen EP. Measurement and evaluation in psychology and education. 5th ed. New York: Macmillan Publishing Co, Inc; 1991.

[29] Plichta SB, Kelvin EA. Munro’s statistical methods for health care research. 6th ed. Philadelphia: Wolters Kluwer Health/Lippincott Williams & Wilkins; 2013.

[30] Sharif Nia H, Ebadi A, Lehto RH, Mousavi B, Peyrovi H, Chan YH (2014). Reliability and validity of the persian version of templer death anxiety scale-extended in veterans of Iran-Iraq warfare. Iran J psychiatry Behav Sci..

[31] Çokluk Ö, Koçak D (2016). Using Horn’s parallel analysis method in exploratory factor analysis for determining the number of factors. Educational Science: Theory & Practice..

[32] Collett LJ, Lester D (1969). The fear of death and the fear of dying. J Psychol..

[33] Colton D, Covert RW (2007). Designing and constructing instruments for social research and evaluation..

[34] Hair JF, Black WC, Babin BJ, Anderson RE. Multivariate data analysis. 7th ed. USA: Pearson Prentice Hall; 2010. p. 691.

[35] Hancock GR, Mueller RO. Rethinking construct reliability within latent variable systems. Lincolnwood: Struct Equ Model Present and Futur; 2001. p. 195–216.

[36] Ebadi A, Zarshenas L, Rakhshan M (2017). Principles of scale development in health sciences.

[37] Wu CY, Chuang LL, Lin KC, Lee SD, Hong W (2011). Responsiveness, minimal detectable change, and minimal clinically important difference of the Nottingham Extended Activities of Daily Living Scale in patients with improved performance after stroke rehabilitation. Arch Phys Med Rehabil..

[38] Polit DF (2015). Assessing measurement in health: Beyond reliability and validity. Int J Nurs Stud..

[39] Mokkink LB, Terwee CB, Patrick DL, Alonso J, Stratford PW, Knol DL (2010). The COSMIN checklist for assessing the methodological quality of studies on measurement properties of health status measurement instruments: An international Delphi study. Qual Life Res..

[40] Esposito Vinzi V, Chin WW, Henseler J, Wang H (2010). Handbook of partial least squares: Concepts, methods and applications.

[41] Mokhtaryan-Gilani T, Ozgoli G, Kariman N, Sharif Nia H, Ahmadi Doulabi M, Nasiri M (2021). Psychometric properties of the Persian translation of maternal postpartum quality of life questionnaire (MAPP-QOL). Health Qual Life Outcomes..

[42] Xiaowen W, Guangping G, Ling Z, Jiarui Z, Xiumin L, Zhaoqin L (2018). Depression and anxiety mediate perceived social support to predict health-related quality of life in pregnant women living with HIV. AIDS Care..

[43] Akbay AS, Tasci-Duran E (2018). How Does Spousal Support Affect Women’S Quality Of Life In The Postpartum Period In Turkish Culture?. Asian Women..

[44] Webster J, Nicholas C, Velacott C, Cridland N, Fawcett L (2010). Validation of the WHOQOL-BREF among women following childbirth. Aust N Z J Obstet Gynaecol..

[45] Rezaei N, Janani F, Sharifi N, Omidi F, Azadi A (2018). Sexual Function and Quality of Life Among Postpartum Women: A Cross-Sectional Study. Int J Women’s Heal Reprod Sci..

[46] Gharibi H, Sheidai A, Rostami CH (2017). The Effectiveness of Parenting Skills Training on Attachment, Perceived Stress and Quality of Life in Mothers of Preschool Children. J Heal and Care..

[47] McNamara J, Townsend ML, Herbert JS (2019). A systemic review of maternal wellbeing and its relationship with maternal fetal attachment and early postpartum bonding. PLoS One..

[48] Triviño-Juárez JM, Nieto-Pereda B, Romero-Ayuso D, Arruti-Sevilla B, Avilés-Gámez B, Forjaz MJ (2016). Quality of life of mothers at the sixth week and sixth month post partum and type of infant feeding. Midwifery..

[49] Kenyhercz F, Kató S, Nagy BE (2021). Health-related quality of life of premature infants at 2 years in relation to breastfeeding and maternal emotional state: a retrospective cohort study. Early Child Dev Care..

[50] Siebert HM. The Transition to Parenthood: Change, Challenges, and Marital Satisfaction. USA: University of South Dakota; 2011.

[51] Xuereb RB, Abela A, Spiteri G (2012). Early parenting–portraits from the lives of first-time parents. J Reprod Infant Psychol..

[52] Symon A, MacDonald A, Ruta D (2002). Postnatal Quality of Life Assessment: Introducing the Mother-Generated Index. Birth..

